# Surface Acoustic Waves to Drive Plant Transpiration

**DOI:** 10.1038/srep45864

**Published:** 2017-03-31

**Authors:** Eliot F. Gomez, Magnus Berggren, Daniel T. Simon

**Affiliations:** 1 Laboratory of Organic Electronics, Department of Science and Technology, Linköping University, SE-601 74 Norrköping, Sweden

## Abstract

Emerging fields of research in electronic plants (e-plants) and agro-nanotechnology seek to create more advanced control of plants and their products. Electronic/nanotechnology plant systems strive to seamlessly monitor, harvest, or deliver chemical signals to sense or regulate plant physiology in a controlled manner. Since the plant vascular system (xylem/phloem) is the primary pathway used to transport water, nutrients, and chemical signals—as well as the primary vehicle for current e-plant and phtyo-nanotechnology work—we seek to directly control fluid transport in plants using external energy. Surface acoustic waves generated from piezoelectric substrates were directly coupled into rose leaves, thereby causing water to rapidly evaporate in a highly localized manner only at the site in contact with the actuator. From fluorescent imaging, we find that the technique reliably delivers up to 6x more water/solute to the site actuated by acoustic energy as compared to normal plant transpiration rates and 2x more than heat-assisted evaporation. The technique of increasing natural plant transpiration through acoustic energy could be used to deliver biomolecules, agrochemicals, or future electronic materials at high spatiotemporal resolution to targeted areas in the plant; providing better interaction with plant physiology or to realize more sophisticated cyborg systems.

The Plantae Kingdom is beginning to intersect with electronic systems^1^ and nanotechnology^2^,^3^ to open up potential opportunities in plant science, energy harvesting, green electronics technology, and new tools for the agriculture industry. Recent efforts have attempted advanced biological interactions to induce physiological changes inside of living plants or trees. Examples include nanoparticles to augment photosynthesis^4^ and organic transistors for energy storage and sensors^5^. In order to establish a generic technology platform to record and regulate plants, it is important to find safe, effective, and complementary technologies that directly sense or affect the various physiological mechanisms and natural processes with high selectivity and spatiotemporal resolution.

Regulating the properties of the plant vascular system is among the most straightforward ways to affect plant physiology or to control the delivery of materials, such as phytohormones. Micron-sized tubular channels comprise bundles of xylem and phloem that deliver water, nutrients, sugar, and chemical signals throughout the plant. Water is transported upwards through the plant via differences in solute pressure and capillary action driven by surface tension provided along the xylem. Plants naturally transpire such that water evaporates through stomata pores in the leaves that cause more water to draw up from the roots. Transpiration rates depend on numerous factors including temperature, wind, humidity, light, soil water level, and leaf physiology^6^. In addition to water uptake, many ionic species and nutrients are transported via the phloem, which is a multidirectional vascular system.

In this paper, we leverage surface acoustic waves (SAW)^7^ as a simple method to directly affect the vascular system by rapidly increasing water evaporation (consequently solute delivery) with spatial and temporal precision. This is done by accelerating evaporation in leaves through acoustic vibrations, similar to mechanisms used to drive fluids in cellulose-based microchannels^8^. SAW are ultrasonic vibrations induced by interdigitated electrodes located on top of a piezoelectric substrate (e.g. lithium niobate, LiNbO_3_). An alternating current at the resonant frequency of the electrodes (typically MHz-GHz) actuates mechanical waves (λ = 1–100 μm) that propagate along the surface with concentrated inertial force. The waves can interact with media in physical contact with the surface to mix, translate, and atomize fluids, or to manipulate micro/nanoscale objects^9^. SAW microfluidics have recently expanded to biological applications and lab-on-chip systems^10^,^11^,^12^,^13^ due to simple fabrication, reusability, and low input power (typically <500 mW). Furthermore, the high frequency and low power of SAW reduce the chance of shearing cell membranes or damaging biomolecules^11^,^12^,^13^.

Several notable works related to this study include: microfluidic platforms deployed for various plant studies^14^,^15^; ultrasonic energy to evaluate post-harvest fruit quality^16^; and leaf-templated microfluidic channels^17^. But plants contain their own natural microfluidic network in their vascular bundles directly capable of affecting physiology. SAW offers a rich complementing technology to manipulate fluid or particle transport inside plants. Acoustically controlled transpiration lays the groundwork for more complex experiments that locally deliver materials or affect physiology for e-plants, plant biology, or agricultural purposes.

## Results

**Figure 1:**
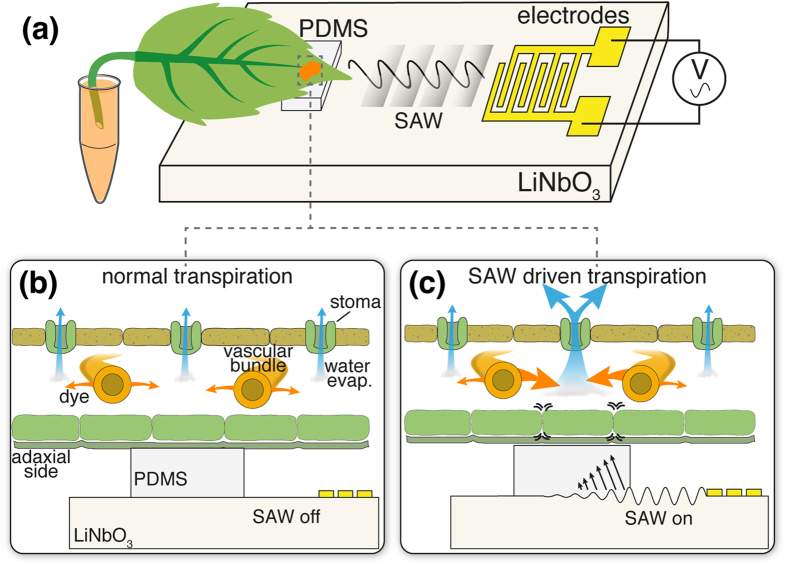
Leaf on SAW device to increase transpiration. (**a**) High frequency mechanical waves produced on the SAW substrate are coupled into a cut leaf submerged in dye. (**b**) A cross section of a leaf without SAW energy, water (blue arrows) evaporates normally through stomata causing a steady flow of water and dye (orange arrows) entering from the vascular bundle. (**c**) SAW is applied and the local acoustic energy increases water evaporation, thereby causing more dye to concentrate at the spot, while the rest of the leaf continues at slower transpiration.

Interdigitated electrodes were patterned on top of the piezoelectric device to produce a ca. 10 MHz surface wave 2 mm wide. A piece of polydimethylsiloxane (PDMS) (3 × 6 × 0.5 mm) was placed on top of the SAW in front of electrodes as illustrated in Fig. 1a. PDMS has known acoustic damping effects^18^ due to its elastic nature that results in (non-directional) in-plane and cross-film displacement gradients that are sources for radiating waves into the film^19^. Although not typically ideal for SAW energy transfer, PDMS offers is a non-liquid couplant with a conformable interface with the leaf that provided the most consistent results. Water, glycerol, and white petrolatum were also tried as a couplant, but the liquid disperses or evaporates. The PDMS thickness was chosen in order to sufficiently raise the leaf above the SAW substrate, while remaining thin enough to couple acoustic energy. The SAW device was connected to an amplified AC power supply and placed on top of a thermoelectric cooling device to ensure that the device was maintained at room temperature throughout the experiments.

Leaves were cut from a *Rosa floribunda* (garden rose) and the stem was submerged in dye (food coloring or fluorescein). The leaf was immediately placed adaxial (top) side down on the PDMS/SAW device. When the SAW is off, the leaf undergoes normal transpiration and evaporates water residing inside of the leaf through the stomata, thereby drawing more dye in from the veins, shown in the cross-section in Fig. 1b. When the SAW is applied (Fig. 1c), the mechanical waves emitted from the interdigital transducer encounter the PDMS couplant that dissipates acoustic energy vertically into the leaf. The acoustic energy disturbs the water and air residing in the spongy mesophyll thereby locally increasing the transpiration rate through the stomata, similar to effects of SAW driven transpiration in cellulose microchannels^8^,^20^. As water evaporates, the dye concentration increases faster at the location of the SAW.

**Figure 2:**
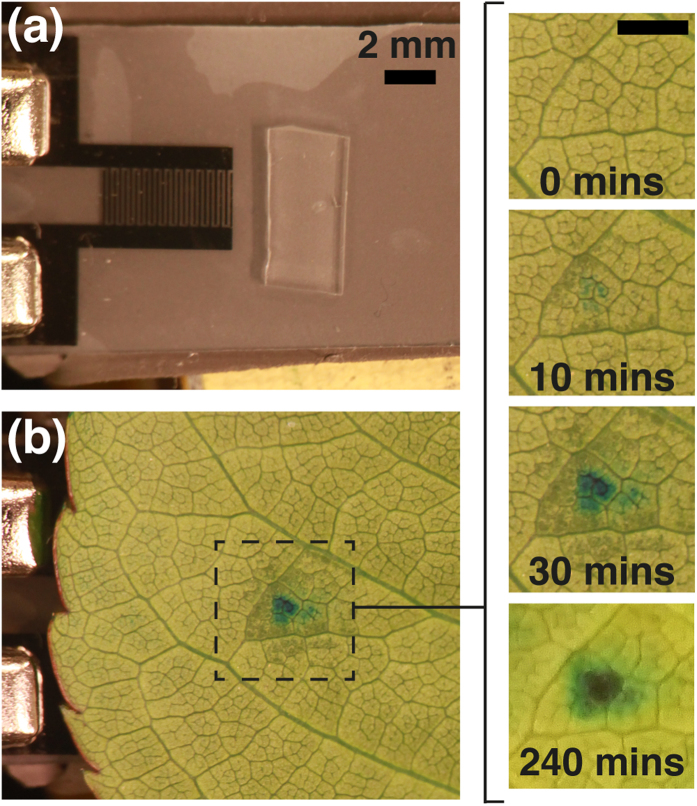
Local delivery of coloring dye with SAW. (**a**) SAW device with electrodes produce 10 MHz waves that encounter a PDMS couplant. (**b**) A leaf is placed on top of the active device and blue dye appears within 30 minutes.

In the first experiment, the leaf stem was submerged in blue food coloring as demonstrated in Fig. 2. A sinusoidal peak-to-peak voltage (100 V_pp_) was applied to the electrodes and images were taken every minute. A picture of the device without the leaf is shown in Fig. 2a. The leaf is placed on top of the active device and blue dye is visible within 10 minutes, shown in Fig. 2b. Dark spots occurred around the site (not at the site) of SAW actuation. The dark spots possibly suggest that excess fluid is mobilized along the outer periphery of the SAW within the leaf proximate to compensate for the rapid evaporation, shown in close up images of Fig. 2b. Within 30 minutes the blue spot is concentrated and measures approximately 2 mm in diameter (the width of the wave). The blue spot remains at the site, and darkening around the site fades to normal color after a few hours; transpiration continues throughout the entire leaf if remained in the dye (see Supplemental Figures S1 and S2). During the experiment, the rest of the leaf exhibited a fainter blue color, indicating normal transpiration was occurring. There was no evident damage at the site on either side of the leaf except when the voltage of the SAW was raised above 100 V_pp_, resulting in shearing of the cuticle and upper epidermis cells (see Supplemental Figures S2 and S3).

**Figure 3:**
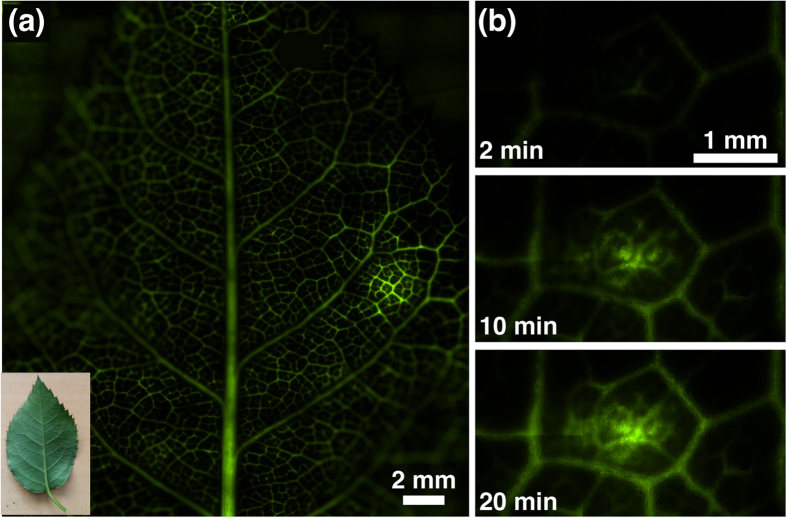
Local delivery of fluorescent molecule with SAW. (**a**) A leaf (inset) fluorescently imaged after 20 minutes of receiving SAW (100 V_pp_). (**b**) A close up of a time-lapse image of the SAW (100 V_pp_) area for 2, 10, and 20 min showing increased delivery to the spongy mesophyll.

In order to observe fluid transport from the veins, the experiment was repeated with fluorescent dye, shown in Fig. 3. The stem of the leaf was placed in fluorescein (1 mM) and SAW was applied to an area on the leaf for 20 minutes. The experiment was repeated for varying voltages (63, 82, or 100 V_pp_) on different leaves. Fluorescein is present in the large vascular bundles within 2 minutes and then the dye begins to slowly enter the area between the veins (spongy mesophyll), which accumulates and integrates over time as water evaporates with transpiration. Figure 3a is an image of the entire leaf after 20 minutes showing that the fluorescence intensity is much greater at the site where acoustic energy was delivered (excluding vascular bundles). Figure 3b shows a series of close up images of a SAW site at 2, 10, and 20 mins indicating delivery to the spongy mesophyll from the veins.

**Figure 4:**
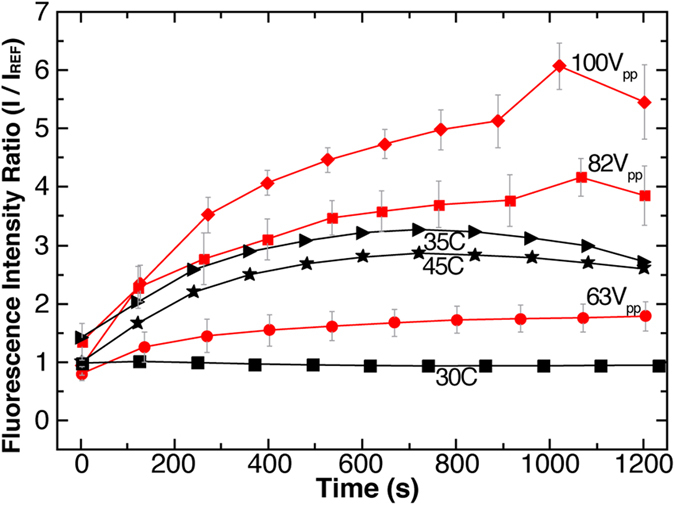
Analysis of fluorescent study. Fluorescence intensity (I) for ratio relative to the reference (I_ref_) with respect to time. Red lines indicate SAW driven evaporation and black lines are heat-assisted evaporation, for different power and temperature.

The red lines in Fig. 4 plot the ratio of the fluorescent intensity for each power setting, where the ratio is determined to be the average intensity of the SAW spot (I) divided by the average intensity of a reference spot (I_ref_) on the same leaf. Since each leaf transpires at slightly different rates, I_ref_ was taken to be a non-SAW spot of the same size on the same leaf for each frame (see Supplemental Figure S4). This fluorescence ratio is thus an approximation of the relative transpiration rates between the SAW spot and the reference spot. The actual transpiration relation is (dI/dt)/(dI_ref_/dt) (Supplementary Figure S5). The lowest power (generated by 63 V_pp_) resulted in 1.25–1.5x increase in the fluorescence ratio. As the voltage increases (82 V_pp_), the transpiration ratio increases 2x higher by 120 s, and 4x higher at 1050 s, respectively. At the highest voltage (100 V_pp_) the SAW increases the fluorescent intensity 3.5x higher, after 270 s, and upwards to 6x, after 1030 s, respectively. Deviation from 1x at t = 0 s are due to spot-to-spot intensity variation before application of SAW.

It is important to note that the same effect is possible with lower power simply by using a thinner or less-dampening medium (such as water), or choosing a more elaborated design of the interdigitated transducer (focused transducers). A couplant is necessary, as little to no effects are observed for a leaf placed directly on top of the piezoelectric substrate (see Supplemental Figure S6).

Heat is another possible method of increasing transpiration. Heat was applied locally to the leaf using a thermoelectric substrate and fluorescent analysis was done in a similar manner as in the SAW experiments. The black lines in Fig. 4 plot the results of temperature effects on the leaf (30, 35, and 45 °C). The results at 30 °C had little to no effect on transpiration. At 35 °C the transpiration rate increases to reach a maximum of 3.3x above the reference, however after 720 s, the rate plateaued and began to decrease. At 45 °C, the transpiration rate plateaued to 2.9x above the reference, suggesting that a higher temperature adversely affects the rate. As compared to heat, SAW offers more than twice the transpiration rate and is more controllable over time.

The effectiveness of the electrically-modulated SAW transpiration can also be seen via a qualitative approximation of the water delivered per area. Several leaves and their solution were weighed after 20 minutes of normal (non-SAW) transpiration to measure how much water was evaporated per area. The leaves were then imaged and sampled to establish a fluorescein amount to fluorescent intensity correlation. It was found that a normal rose leaf transpires approximately 30–60 μg mm^−2^ hr^−1^ of water where the SAW driven transpiration delivers water at a rate of 180–210 μg mm^−2^ hr^−1^. The amount of solute delivered is contingent on many variables, including the type of leaf, but nevertheless these values may be useful to determine possible local delivery of drugs or hormones for future plant studies.

## Discussion

In this work, acoustic energy is used for the first time to control transpiration in plants upwards of 6x higher than normal transpiration rates. The results were faster and more controllable than using heat to deliver materials via increased transpiration to a specific site in a leaf. Based on our results, it appears the leaf operates in different modes with heat-based versus SAW-based transpiration. Temperature (as well as other factors such as solar flux, humidity, CO_2_ levels) can exponential increase stomata resistance (r_s_) and diminish transpiration if the leaf temperature surpasses a critical value in the leaf (T_l_ > T_C_)^6^. As a result, the transpiration rate plateaus and decreases after some time. The mechanism of SAW-based transpiration, on the other hand, appears to disturb and increase water potential of the mesophyll (ψ_l_) thereby driving water flux forward through the stomata. In the case of the SAW, however, the r_s_ appears relatively unaffected. We believe that r_s_ may remain low since the water potential on the outside of the leaf near the guard cells (ψ_S_) is unchanged, thus not reaching a critical potential (ψ_C_) needed to induce stomata closure. (see Lynn *et al*.^6^ for a complete model and discussion of transpiration parameters). As a result, SAW is unhindered during transpiration.

While most of the experiments were performed with a 2 mm wide wave, additional experiments demonstrated that spot sizes ranging from 1 to 6 mm also increased the delivery and transpiration rate (see Supplemental Figure S7). Larger spots may be possible with a larger substrate. In order to see the effects of frequency, a 20 MHz SAW device was used, however, it did not seem to have a notable effect on the transpiration (see Supplemental Figure S7b). It is known that higher frequencies have a shorter attenuation length^21^ therefore would require a larger operating power (or optimized couplant). Furthermore, the electrode feature size for frequencies beyond 20 MHz are difficult to fabricate with printed lithography masks.

Additional experiments were done on plants with waxy leaves (*Epipremnum aureum*) which have significantly slower transpiration rates. SAW was found to increase fluorescent delivery upwards of 15x higher than the reference (see Supplemental Figures S8 and S9). Initial experiments were also done on branches with 3 leaves to show that this technique is capable on larger plants (see Supplemental Figures S10 and S11). While still preliminary, these results demonstrate the potential for this technique to expand to rooted systems.

Delivery of solute from rooted systems have direct applications for smart delivery systems^2^, which have been proposed in plants by using engineered nanoparticles for “time-controlled, target-specific” agrochemicals^3^, medicinal purposes^22^, drug release^23^, or genomics^24^. A combination of phyto-nanotechnology with acoustic energy could allow highly spatiotemporal delivery. In addition, there remain many questions about ion delivery and storage in plants, such as Ca^2+^ for example^25^, that play important roles in signaling. Local manipulation or release using acoustic energy could offer beneficial results under controlled plant studies from targeted, dynamic increase in plant signaling compounds (*e.g.*, hormones).

SAW could eventually be used to deliver water soluble electronic material to pinpoint e-plant fabrication inside of plants. We show preliminary results delivering an (3,4-ethylenedioxythiophene) EDOT-based trimer^5^ with SAW to plant leaves in Supplemental Figure S12. However, unlike the fluorescein, sufficient trimer did not reach the active area or perhaps prematurely aggregated or polymerized in the vascular system, thus clogging the leaf. Further investigation is required to overcome the challenges of the molecule size and premature cross-linking in order to have a better understanding of the mechanisms of electronically conducting polymers and small molecules inside of plants.

Based on this initial exploration of SAW applied to plants, we recommend some future directions and possibilities for the technology. The first step would be to explore the minimum resolution of SAW-driven transpiration. We attempted a 1 mm delivery, but the absolute resolution is limited to the SAW wavelength. Higher frequencies generate micron-sized (cellular or capillary-sized) waves, but the challenge will be to couple the energy to the desired location without dissipation or attenuation. High resolution most likely will require additional methods, such as cooling the leaf to decrease overall transpiration.

In some cases, it may be desired to create different patterns with SAW other than a spot. The leaf could be physically moved during acoustic actuation, but this would inevitably decrease the contrast ratio. Another method would be to alter the shape of the couplant. The problem with this is the wave attenuates as it encounters the couplant, thus would result in non-uniform delivery for areas that are farther away from the incident wave. But a relatively simple solution is to incorporate multiple SAW transducers to ensure equal SAW intensity is received on all sides.

Beyond the ability to control transpiration, surface acoustic waves have advanced to more powerful techniques that directly affect microfluidic channels. Acoustic tweezers^26^,^27^ and Standing SAW (SSAW) produce standing pressure gradients with two or more intersecting waves. SSAW has been used to sort, mix, pump, filter, and create well defined pressure gradients in bulk fluids, or to sort/manipulate microparticles or cells^9^,^28^,^29^. Extension of these well-established techniques into xylem/phloem channels could further tailor specific delivery to specific cells or even organelles by acoustic energy in the stem. Such SSAW applications could be facilitated by proof-of-principle in simple plant system such as pine needles (single xylem/phloem channel) or in single vascular tubes of a leaf. Expanded applications could include, for example, a SSAW in a xylem channel in the stem to create a pressure gradient causing nanoparticles to be diverted or filtered at branch nodes, using a similar technique as described by Destgeer *et al*.^9^ Combined with SAW-transpiration, a future matrix-addressed system could “demultiplex” particles or fluids that are delivered to the leaf from the roots, depending on the desired application.

We see acoustic energy as a powerful tool for future e-plant and agro-nanotechnology. We expect this technique to be explored further, either as a complimentary tool for studying natural processes or for plant fluidic manipulation that results in greater control of physiology. As interaction with plants become more necessary, the tools of well-established microfluidic systems will be increasingly useful to fabricate electronic systems inside of living plants or develop advanced agricultural techniques.

## Methods

### SAW Fabrication

Lithium Niobate wafers were purchased from The Roditi International Corporation. The wafers (100 × 0.50 mm, 127.68° y-cut) were scribed to a size of 15 × 25 mm and cleaned with acetone, ethanol, DI water, and dried with nitrogen. Several photolithography masks were designed and screen printed (Coated Screens Scandinavia AB) to pattern the electrodes. The 10 MHz device had interdigitated electrodes with a 2 mm wide aperture and 25 fingers with a spacing of d = 100 μm (f ≈ ν_saw_/λ = ν_saw_/2d), where ν_saw_ = 3990 m/s the propagation in the x-direction of the substrate and λ is the wavelength of the propagating wave. After the substrate was cleaned, positive photoresist (S1818) was spincoated on the polished side at 3000 rpm for 30 s and baked at 90 °C for 1 minute. The lithography mask and the resist were exposed and removed with developer MICROPOSIT^®^ MF-321. Afterwards, Ti and Au electrodes were deposited in a thermal evaporation system (Balzers BA510) to a thickness of 4 and 70 nm, respectively, and then washed in acetone to remove the photoresist mask.

### SAW and heat experiments

A Waveform Generator (Agilent 33250A) amplified through RF solid state amplifier (IFI M75, D+M Systems and Test, 75 W, 48 dB) generated a SAW wave at a resonance frequency of 9.89 MHz. An oscilloscope verified the amplified output voltage but was not connected during the experiment. PDMS was mixed and cured in a 1:10 ratio using a standard procedure. The SAW substrate was placed on top of a Thermoelectric Peltier substrate (PE-071-14-15-S Laird, Elfa Distrelec) with a heat conducting film (GP3000S30 0.25mm) in between the elements. The leaf was placed adaxial side on the PDMS with tweezers weighing the down leaf to ensure it was completely touching the PDMS but not the SAW device.

A power supply connected to the thermoelectric substrate cooled the substrate, typically requiring 0–1.5 W, providing a temperature range between 10–20 °C (measured with Agilent u1271A), and adjusted accordingly to ensure the SAW device maintained room temperature at all times. Humidity was 30–40% for all experiments. Heat experiments were done in the same way, except the thermoelectric substrate was heated. The leaf was placed on a local area which was heated on the leaf.

### Plant conditions and preparation

A rose plant (*R. fluoribunda*) was purchased from a local flower shop and cared for inside at about 40% humidity with regular watering and direct afternoon sunlight. For each experiment, a leaf was excised, rinsed, dried, and then recut under water. The stem was immediately placed in a vial containing dye and the vial wrapped in film to prevent evaporation.

### Optical measurements

Blue food coloring (Dr Oetker) was purchased and diluted 1:2, dye to city water. Imaging was done with a Canon 5D m2 with a macro lens every 1 minute.

### Fluorescent microscopy

A 0.1 M stock sample of fluorescein (in 10% methanol and 90% isopropanol) was further diluted to obtain 1 mM of dye in city water. Images were taken using a Microscope Nikon Eclipse Ni-E (BergmanLabora AB) through a single-band filter cube (FITC, 480 nm excitation, 525 nm emission). Imaging and SAW started immediately after the stem was submerged in dye and placed on the device. A large area of the leaf was imaged (typically 8 × 10 images, resulting in a 90 s lag time to capture) every 2 minutes for 20 minutes with a 200 ms exposure time.

### Image analysis

Images were automatically stitched together and analyzed using the NIS software. The average fluorescence intensity was measured with a region of interest (ROI) circle size of 2.54 mm^2^ for each time interval. Three ROI reference points were taken of the same size and averaged together each time interval, see Supplemental Figure S4. Each power setting (and heat setting) was repeated 4–5 times and averaged together. Error was calculated using the standard error of the mean for each SAW setting.

## Additional Information

**How to cite this article:** Gomez, E. F. *et al*. Surface Acoustic Waves to Drive Plant Transpiration. *Sci. Rep.* **7**, 45864; doi: 10.1038/srep45864 (2017).

**Publisher's note:** Springer Nature remains neutral with regard to jurisdictional claims in published maps and institutional affiliations.

## Supplementary Material

Supplemental Figures
