# Supplementary material for: Surface Acoustic Waves to Drive Plant Transpiration
Source: Sci Rep. 2017 Mar 31;7:45864. doi: 10.1038/srep45864 (PMC5374464; doi:10.1038/srep45864)
Supplement: Supplemental Figures [file srep45864-s1.doc]

Eliot F. Gomez, Magnus Berggren*, & Daniel T. Simon

*Laboratory of Organic Electronics, Department of Science and Technology, Linköping University, SE-601 74 Norrköping, Sweden*


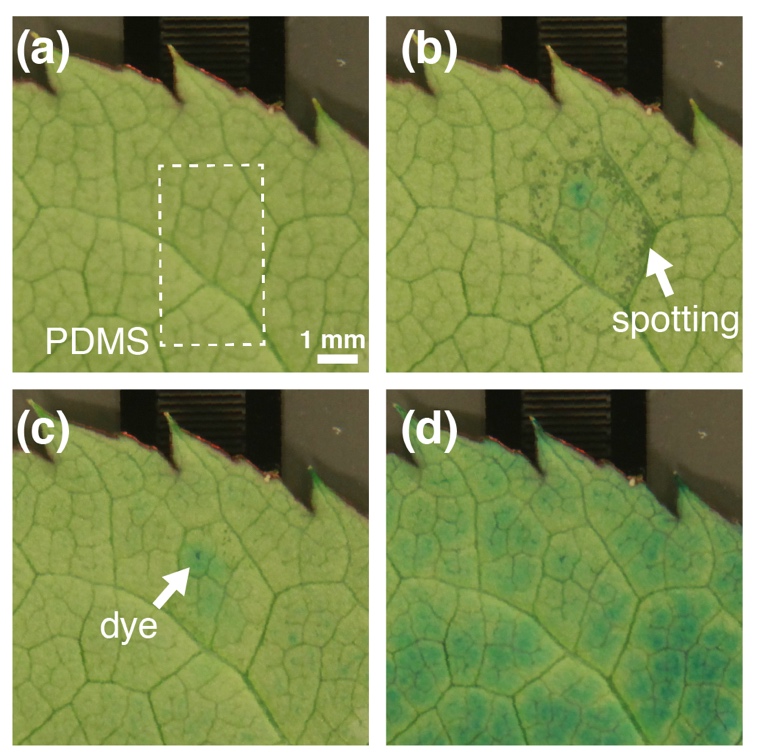


**Figure S1** – (a) A leaf is placed on SAW (63 Vpp). (b) Characteristic spotting occurs around the active area. (c) SAW is removed and the spotting disappears. (d) Transpiration continues on all other areas of the leaf. t=0, 90, 120, 240 min, respectively.


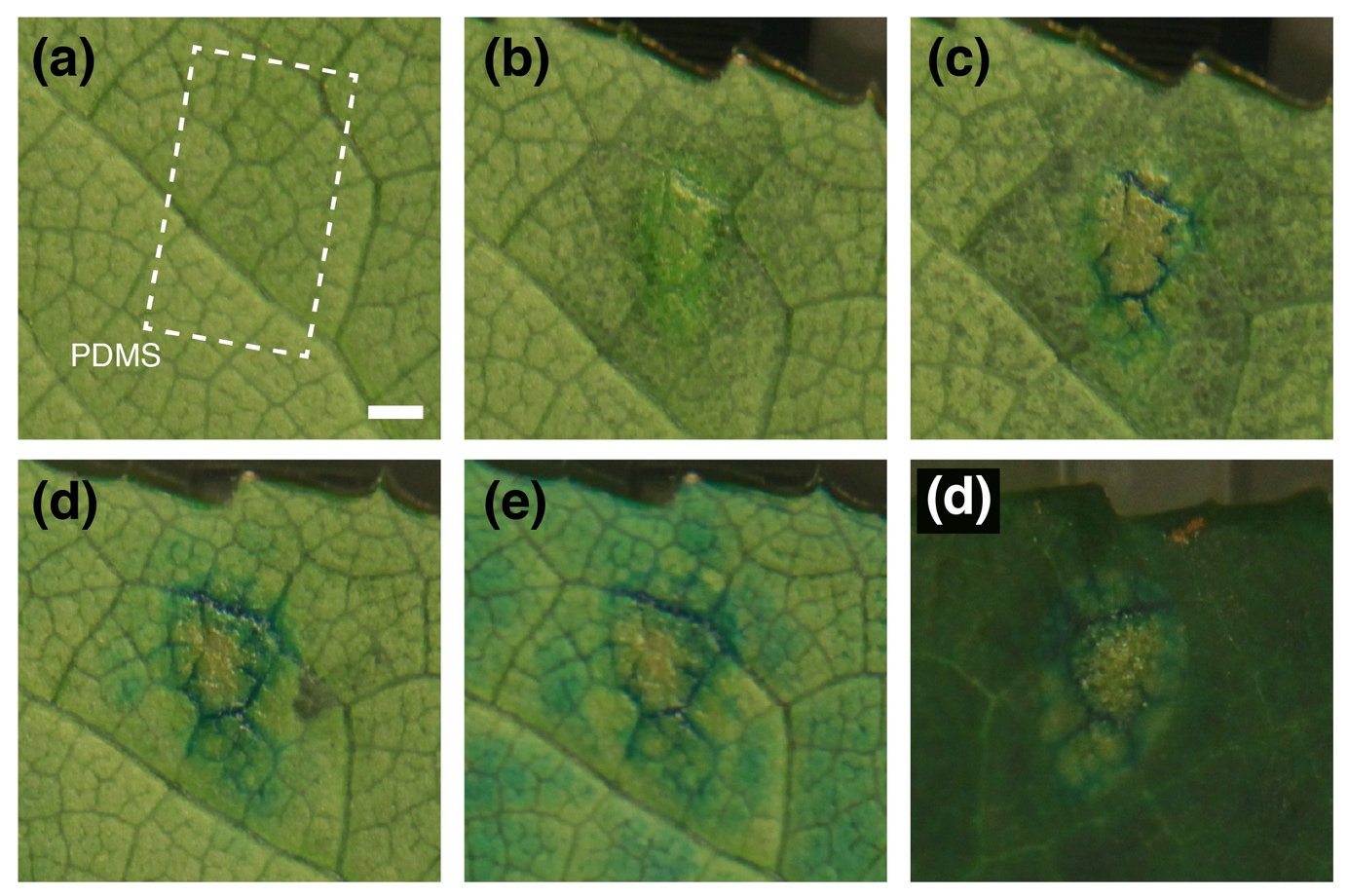


**Figure S2** –(a-e) At high power (125 V_pp_) delivery the area dries out very rapidly and the dye remains concentrated in the veins. t=1, 2, 12, 50, 160 (d) Damage is evident on the adaxial side of the leaf.


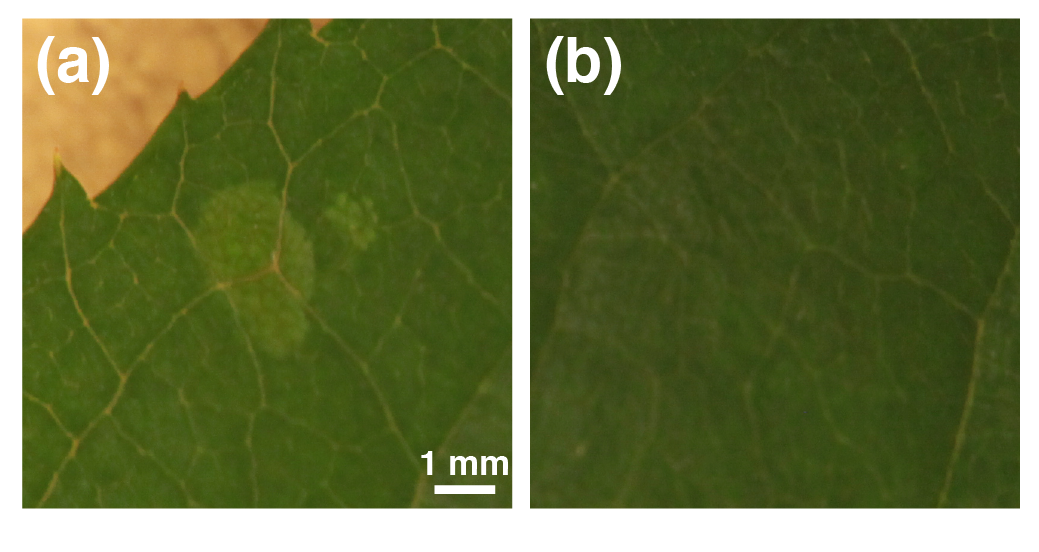


**Figure S3** – (a) Damage occurs at the leaf at high powers >100 V_pp_ on the adaxial side. (b) No damage is evident ≤100 Vpp even exposed for more than 90 min.


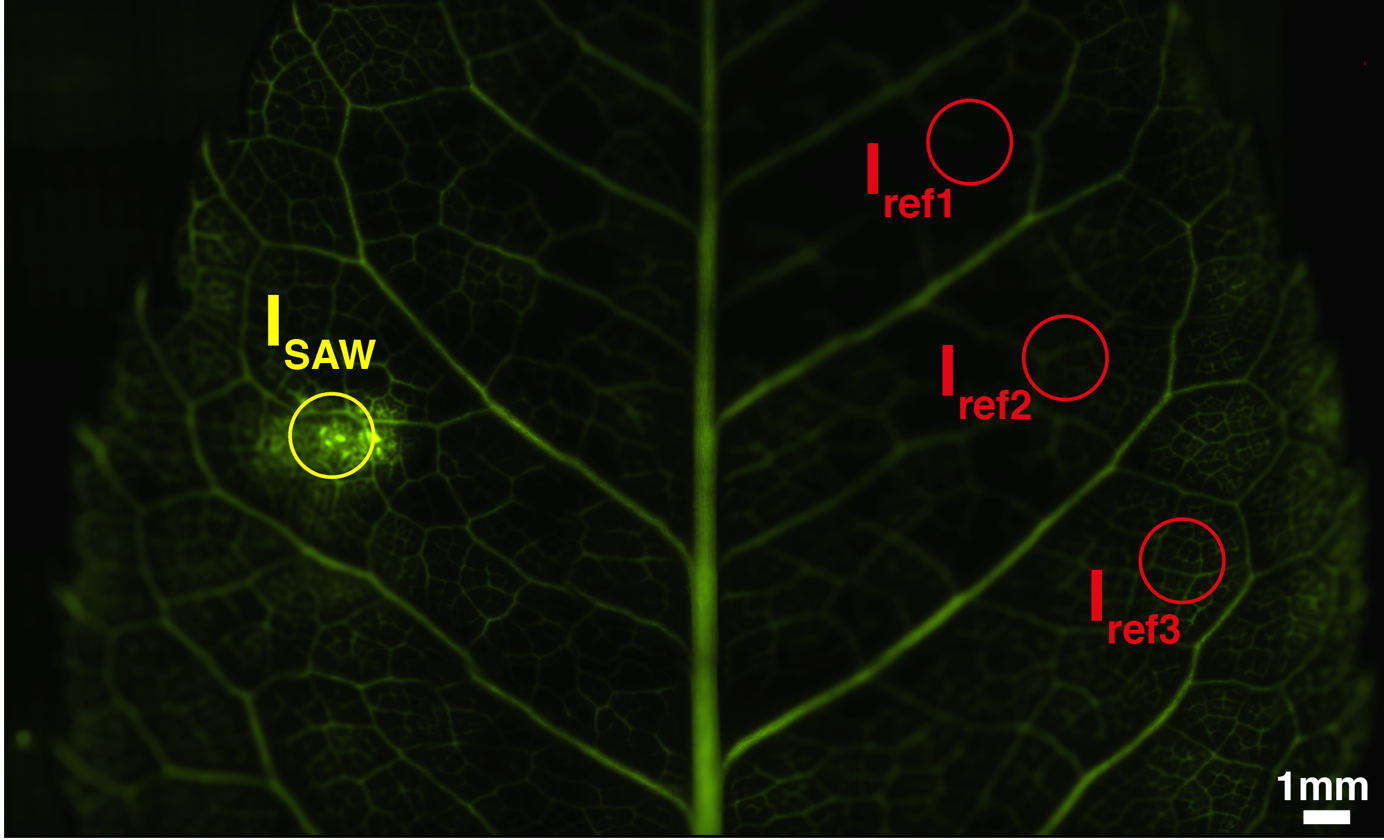


**Figure S4** – An example of a sampling for the fluorescent studies. Three reference points (red) are used on the opposite side of the leaf that did not experience the SAW wave and the average intensities of the circle are averaged together for the reference value. The ratio of the SAW average (green) is then taken over the reference average. Major vascular bundles are avoided.


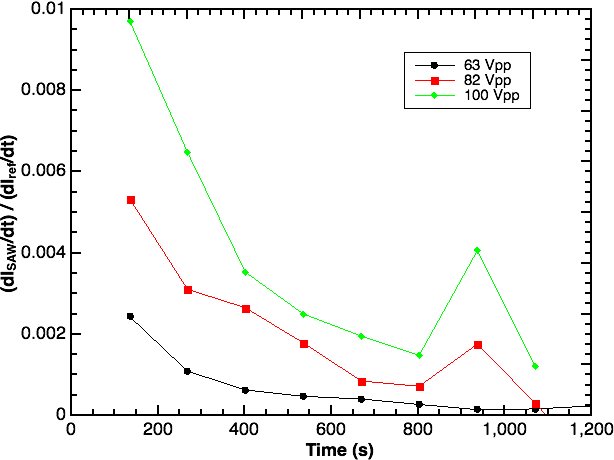


**Figure S5** – Transpiration rate of Figure 4 plotted as the time derivative of the SAW to normal transpiration.


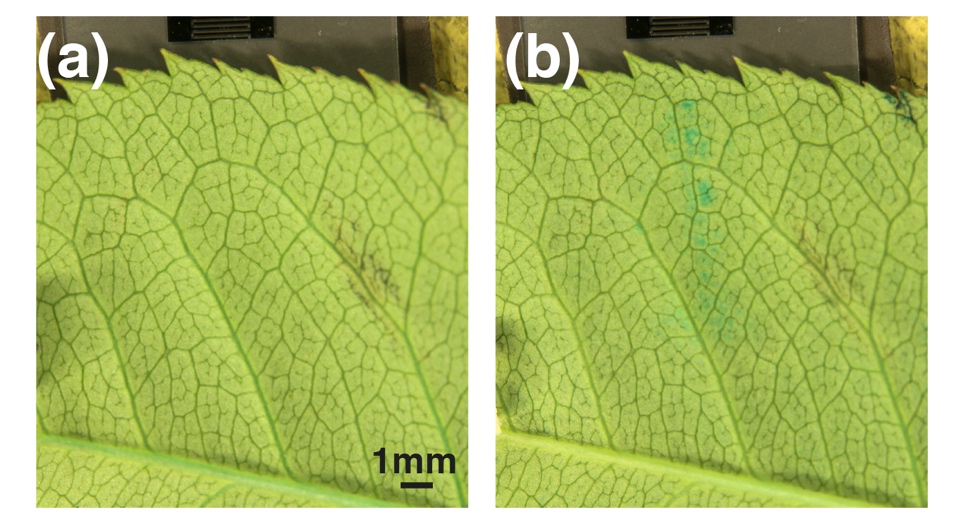


**Figure S6**- a) Leaf in blue dye without PDMS couplant experiencing a SAW wave at Vpp = >100 at t =0 min b) after 50 mins a faint blue dye occurs at the aperture.


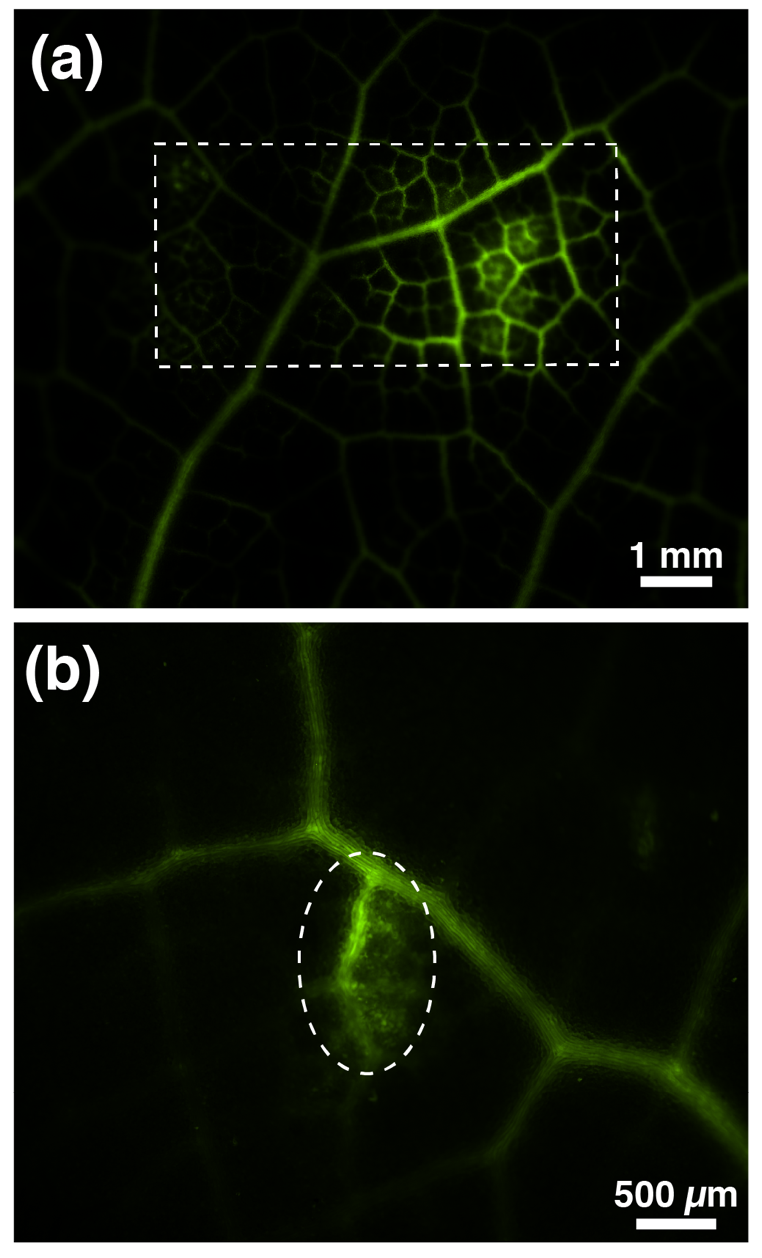


**Figure S7** – (a) Transpiration of a 6mm wide wave at 10 MHz. The dotted line indicates the PDMS couplant. (b) an example of a 1 mm wide wave at 20 MHz. The dotted line is the petroleum couplant underneath the leaf.


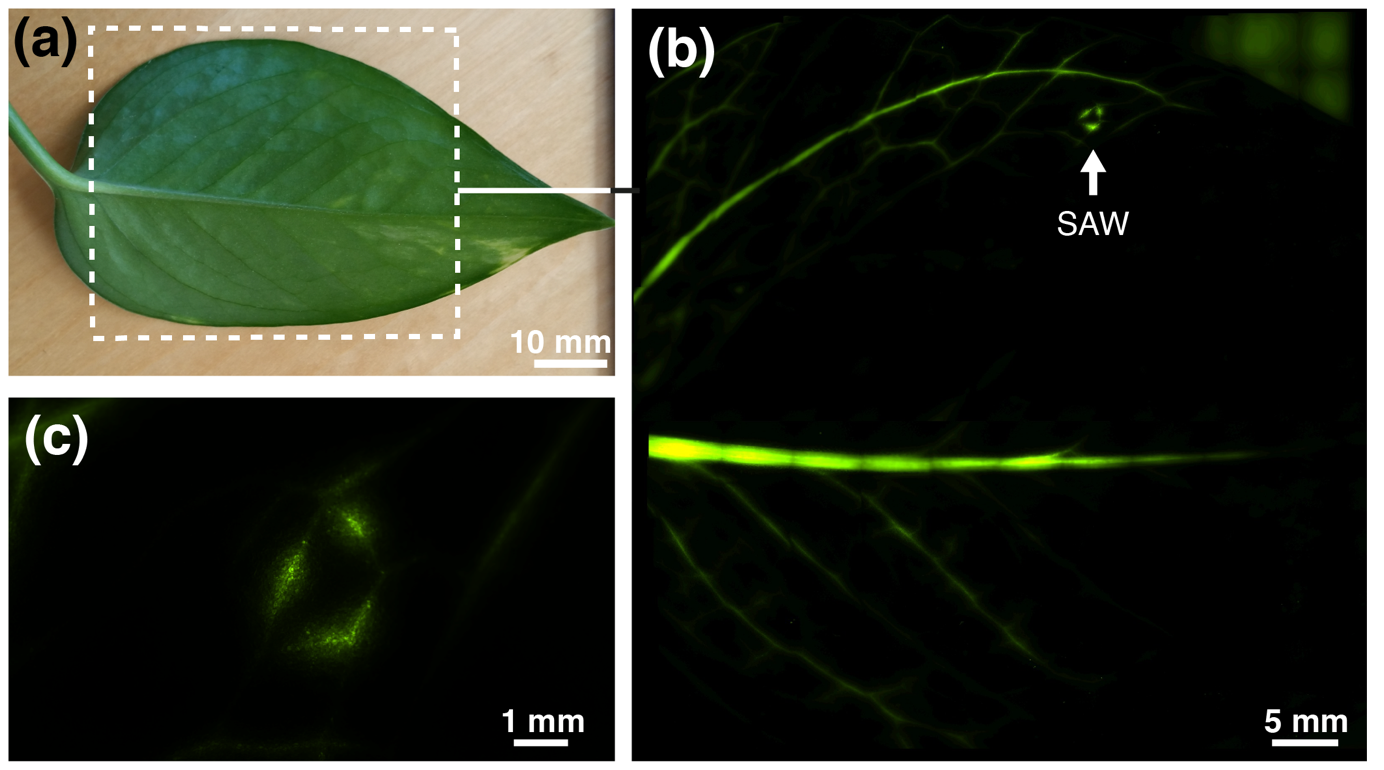


**Figure S8** – SAW on an *Epipremnum aureum* plant has a waxy cuticle that retains water better and has a slow transpiration rate. SAW increases the transpiration upwards of 15x more than normal transpiration.


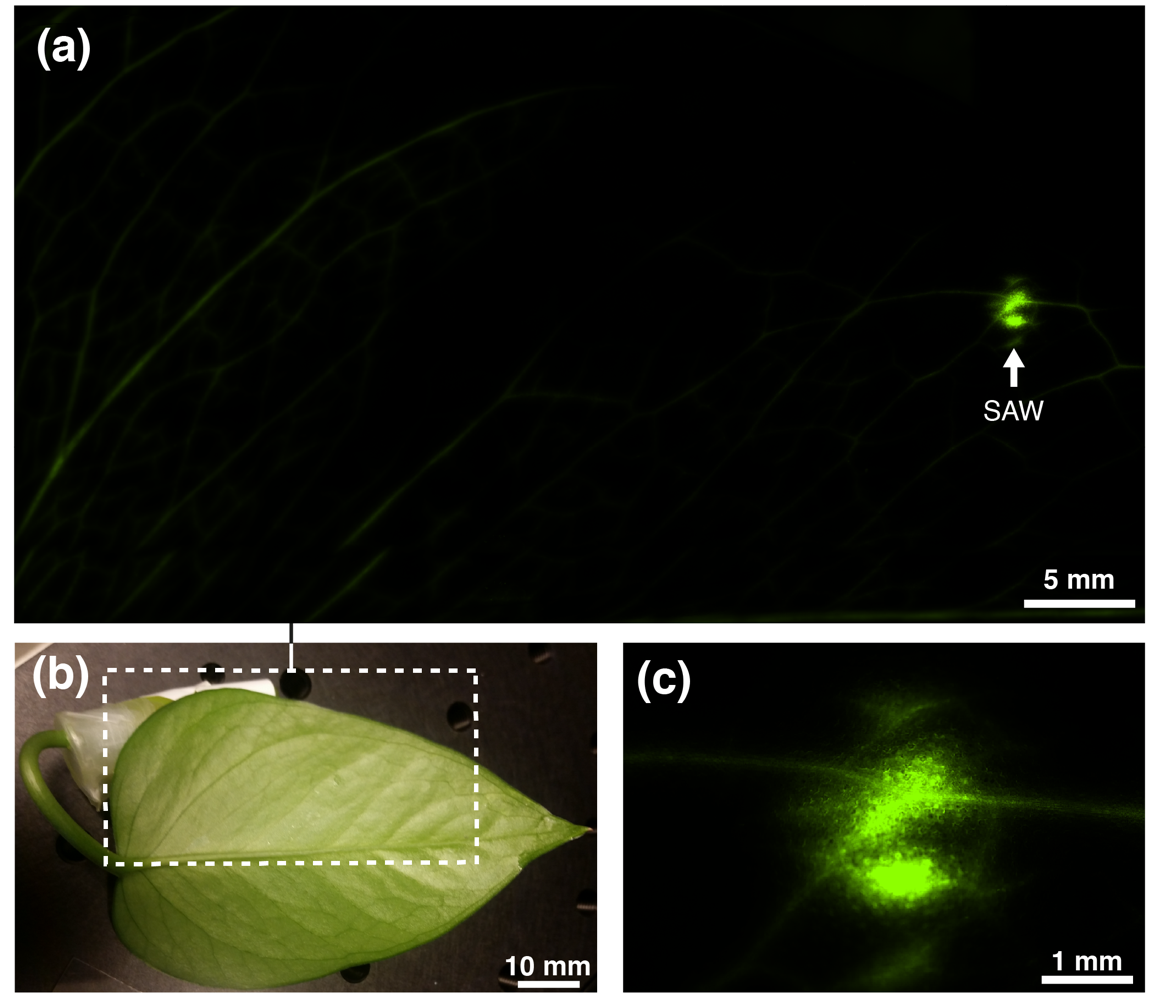


**Figure S9** – Another example of a waxy leaf transpiration using a white petrolatum couplant.


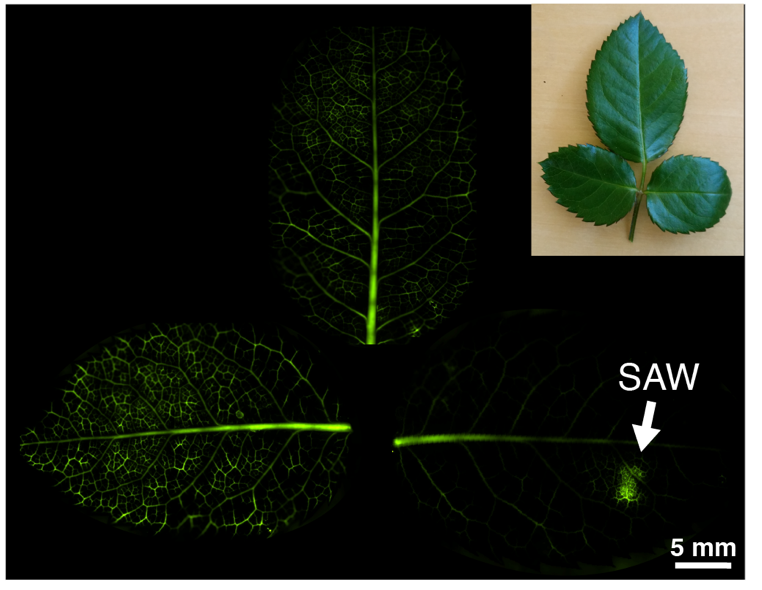


**Figure S10** – Fluorescent imaging of a branch with three leaves. SAW is placed on the bottom right leaf.


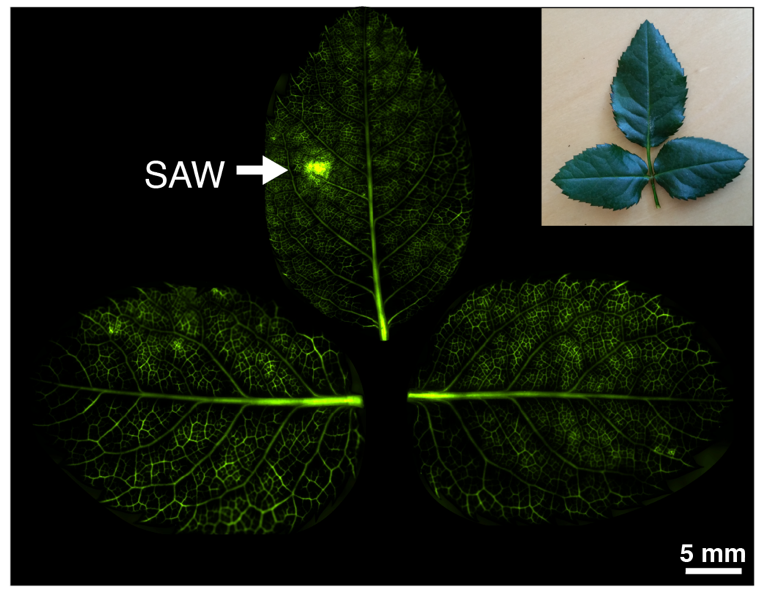


**Figure S11** – Another fluorescent imaging of a branch with three leaves. SAW is placed on the top leaf.


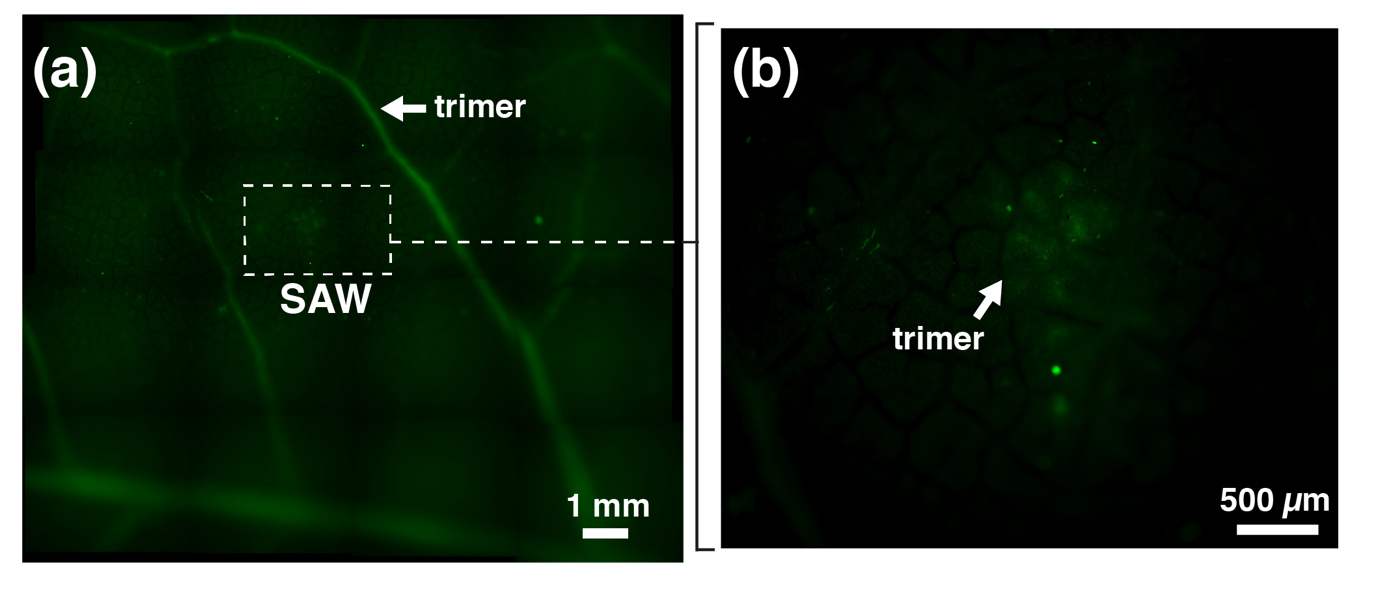


**Figure S12**- Fluorescence of the trimer under SAW 100 V_pp_. (a) Trimer is evident in the veins. (b) Trimer faintly evident in the area SAW. It is likely that the trimer is too large to adequately pass through the veins and crosslink.
